# Adrenal insufficiency in thyroid cancer patients treated with tyrosine kinase inhibitors and detected by ACTH stimulation test

**DOI:** 10.1007/s40618-023-02025-3

**Published:** 2023-02-21

**Authors:** L. Valerio, C. Giani, A. Matrone, B. Pontillo-Contillo, E. Minaldi, L. Agate, E. Molinaro, R. Elisei

**Affiliations:** 1 Department of Clinical and Experimental Medicine, Unit of Endocrinology, University of Pisa, Via Paradisa 2, 56124 Pisa, Italy; 2 Diagnostic and Interventional Radiology Department of Translational Research and New Technologies in Medicine and Surgery, University of Pisa, Via Paradisa 2, 56124 Pisa, Italy

**Keywords:** Adrenal insufficiency, Thyroid cancer, Tyrosine kinase inhibitors, Adverse event, ACTH, Cortisol

## Abstract

**Purpose:**

Advanced thyroid cancer patients treated with tyrosine kinase inhibitors (TKI) can develop several adverse events (AEs), including adrenal insufficiency (AI).

**Methods:**

We studied 55 patients treated with TKI for radioiodine-refractory or medullary thyroid cancer. The adrenal function was evaluated during follow-up by performing serum basal ACTH, and basal and ACTH-stimulated cortisol.

**Results:**

Twenty-nine/55 (52.7%) patients developed subclinical AI during TKI treatment as demonstrated by a blunted cortisol response to ACTH stimulation. All cases showed normal values of serum sodium, potassium and blood pressure. All patients were immediately treated, and none showed an overt AI. Cases with AI were all negative for adrenal antibodies and did not show any adrenal gland alteration. Other causes of AI were excluded. The onset time of the AI, as measured in the subgroup with a first negative ACTH test, was < 12 months in 5/9 (55.6%), between 12 and 36 months in 2/9 (22.2%) and > 36 months in 2/9 (22.2%) cases. In our series, the only prognostic factor of AI was the elevated, although moderate, basal level of ACTH when the basal and stimulated cortisol were still normal. The glucocorticoid therapy improved fatigue in most patients.

**Conclusions:**

Subclinical AI can be developed in > 50% of advanced thyroid cancer patients treated with TKI. This AE can develop in a wide period ranging from < 12 to > 36 months. For this reason, AI must be looked for throughout the follow-up to be early recognized and treated. A periodic ACTH stimulation test, every 6–8 months, can be helpful.

## Introduction

Tyrosine kinase inhibitors (TKI) represent an important therapeutic option for the treatment of locally advanced or metastatic thyroid cancer [1]. Lenvatinib and sorafenib have been approved for the treatment of advanced and progressive radioiodine-refractory (RAI-R) thyroid cancer [2, 3], while vandetanib and cabozantinib have been approved for the treatment of advanced medullary thyroid cancer [4, 5]. Lenvatinib showed interesting results also as salvage therapy in patients with advanced medullary thyroid cancer [6]. They are all multitarget inhibitors, mainly anti-vascular endothelial growth factor receptors (VEGF-R) [7]. This antiangiogenic activity is both a benefit, because it can contribute to the shrinkage of the tumor, and a drawback, since it is the cause of several adverse events (AEs) [8, 9].

Among several well-known AEs, such as hypertension, fatigue, nausea, anorexia, weight loss and diarrhea, several endocrine dysfunctions have been described [10], but never in the studies reporting the data of the official trials with TKI [2–5]. Among the endocrine dysfunctions, adrenal insufficiency (AI) plays an important role in these patients, since it can severely worsen symptoms already present such as fatigue and asthenia.

Cortisol is an important stress response hormone with multiple functions (metabolic, catabolic, anti-inflammatory and vasoactive). As response to stress, the hypothalamus induces an increase of release of adrenocorticotropic hormone (ACTH) by the anterior pituitary gland. If the system is impaired, either at the central or peripheral level, the cortisol cannot increase and the patient likely succumbs to stress [11]. Since AI can be a life-threating AE, it is very important to look for it and immediately treat, since it can have serious consequences if unrecognized.

While the appearance of AI has been reported in patients treated with PD-1/PD-L1 and CTLA-4 inhibitors, only scanty data are present on oncological patients, and in particular on thyroid cancer patients treated with TKI [12–15]. To our knowledge, only Colombo et al. and Monti et al. recently reported this AE in about 50% of very small series of patients treated with vandetanib or lenvatinib for advanced thyroid cancer [16, 17], thus raising the question of whether all thyroid cancer patients undergoing TKI therapies should be monitored for this AE and its early correction.

The aims of this study were to evaluate the prevalence, severity, and the time of onset of clinical/subclinical AI in a series of advanced thyroid cancer patients treated with different TKI. Moreover, we investigated the possible prognostic factors of this AE and the impact of its correction on the health status of these patients.

## Materials and methods

### Study group

Starting from 2017, a total of 55 patients already under TKI treatment were prospectively submitted to an ACTH stimulation test that is considered the gold standard to define the diagnosis of AI [18]. Among them, 20 (36.4%) patients (Group 1) already showed a blunted response to the first, and in this group unique, ACTH stimulus despite the normal cortisol basal levels. According to this result, and in agreement with the AI guidelines [18], these patients were considered already affected by a subclinical AI. However, since they were already under TKI from several weeks or months (mean 52 months, median 41 months, range 8–146 months), we could not establish when they developed this AE. Thus, for the purpose of defining the timing of onset of the AI, we separated these patients from another group of 35 patients (63.6%) (Group 2) who had normal basal levels of cortisol and ACTH, as well as a normal response to the first ACTH stimulus, thus confirming a normal adrenal function at that time, despite the ongoing TKI treatment. These 35 patients underwent at least a second, or more, ACTH stimulation test during their follow-up. The ACTH stimulation test was repeated every 6 months. Based on these results, we divided our study group into two subgroups: patients with (*n* = 29) (Group A) and without hypocortisolism (*n* = 26) (Group B) developed during follow-up.

The included 55 patients were not under glucocorticoid therapy or any other interfering drug (i.e., opioid), since this represented an exclusion criterion. We also excluded alterations of the pituitary axis with an evaluation of the basal hypophysis function.

All patients gave their signed approval to the use of the clinical and biochemical personal data for research and scientific purposes. The study was approved by the local ethical committee.

### Methods

We evaluated the epidemiological, clinical, pathological, biochemical and radiological data of the 55 patients submitted to the adrenal gland function study. The ACTH stimulation test [i.e., short corticotropin test according to the AI guidelines [18]] was performed between 8.00 and 8.30 am by administering 250 µg of ACTH i.m. and measuring cortisol before and 30 and 60 min after the injection (cortisol assay: Access Immunoassay Systems, Beckman Coulter s.r.l., Milan, Italy [normal range: 6.7–22.6 µg/dL]). Serum ACTH was also measured at the same time points (ACTH assay: Immulite 2000, Siemens Healthcare Diagnostics S.A.S., Saint-Denis cedex 2, France [normal range: 5–50 ng/L]). The diagnosis of AI was defined by a blunted peak of cortisol < 18 µg/dL at 30 or 60 min, associated, in some cases, with elevated basal serum ACTH levels [18]. In all patients who developed the AI, we also evaluated the presence of adrenal antibodies (ELISA, RSR Limited, Cardiff, UK).

In agreement with the indication for monitoring the AEs of TKI treatment, serum sodium (normal range: 135–145 mEq/L) and potassium (normal range: 3.0–5.1 mEq/L) values, as well as the blood pressure values, were evaluated at each examination and at the time of the ACTH stimulation test. For the purpose of identifying any possible adrenal alteration such as cortical congestion, hemorrhage, or necrosis potentially responsible of the AI, a radiographic re-evaluation of adrenal glands was performed in all patients who developed the AI by specifically reviewing the computed tomography (CT) scans performed during follow-up for the monitoring of the disease.

The evaluation of fatigue that represents the main symptom associated with AI, but also one of the most frequent AEs of TKI, was performed by using the National Cancer Institute's Common Terminology Criteria for Adverse Events (CTCAE) [19]. This was done in all patients during follow-up and in particular before and after starting the treatment with cortisone acetate in those patients who developed AI.

### Statistical analysis

Pearson’s Chi-squared test and Fisher’s exact test were used to evaluate differences in counts and frequency between groups, as appropriate. Student’s *t* test and Mann–Whitney *U* tests were used to assess differences between groups for continuous variables with Gaussian and skewed distributions, respectively. Data analysis was performed using StatView 4.5 software (Abacus Concepts Inc., Berkeley, CA). *P* < 0.05 was considered statistically significant. Data are presented as mean ± standard deviation or frequency (percentage).

## Results

As shown in Table 1 (left column), patients were prevalently male with a mean age at diagnosis of 49 years and a mean age at the time of TKI initiation of 55 years. A prevalent percentage of MTC was present likely because our center is a tertiary referral center for this type of thyroid tumor. Most patients were under lenvatinib or vandetanib, but a 5.5% were under cabozantinib. Although more than 80% were under their first-line TKI treatment, a not negligible percentage was under the second- or even fourth-line of therapy.

**Table 1:** Epidemiological, clinical and pathological features of all patients together and separated according to the results of the first ACTH stimulation test

	Total pts (*n* = 55)		Group 1 (*n* = 20)*		Group 2 (*n* = 35)**	
	No.	%	No.	%	No.	%
Sex						
Male Female	39 16	70.9 29.1	16 4	80.0 20.0	23 12	65.7 34.3
Age at diagnosis, years						
Mean Median Range	49.2 47.0 13–68		48.8 46.0 34–65		49.4 47.0 13–68	
Age at screening, years						
Mean Median Range	55.2 58 20–73		55.3 56.5 34–69		55.2 59.0 20–73	
Histology						
PTC FTC MTC PDTC	12 11 24 8	21.8 20.0 43.6 14.6	6 4 8 2	30.0 20.0 40.0 10.0	6 7 16 6	17.1 20.0 45.8 17.1
TKI at ACTH test						
Lenvatinib Vandetanib Cabozantinib	30 22 3	54.5 40.0 5.5	12 8 0	60.0 40.0 0	18 14 3	51.4 40.0 8.6
Lines of TKI at ACTH test						
1 2 4	45 9 1	81.8 16.4 1.8	19 1 0	95.0 5.0 0	26 8 1	74.3 22.8 2.9

*Group 1: cases with the first ACTH test already positive (onset time unknown)

**Group 2: cases with the first ACTH test still negative (prospective cohort to establish the onset time of the adrenal insufficiency)

### Group 1: patients with adrenal insufficiency detected at the first ACTH test

Twenty patients (36.4%) of the study group showed a blunted response (cortisol peak—median: 14.4 µg/dL; mean: 14.3 µg/dL; range: 9.5–17.6 µg/dL) to the short ACTH stimulus at the first stimulation test (Group 1). The median and mean time elapsing from the initiation of TKI therapy and the first ACTH test was 41 months and 52.5 months, respectively, but we cannot exclude that the AI was already present in the previous months. However, all cases had a normal basal level of cortisol and in ten cases (50%) the basal ACTH levels were above the maximum normal value (mean 138 ng/L, median 118 ng/L, range 63–336 nl/L). On the basis of our results and according to the AI guidelines [18], both patients with a high value of basal ACTH (i.e., > twofold upper normal limit (ULN) and those with a positive ACTH test independently from the basal ACTH value were considered affected by a subclinical hypocortisolism and immediately treated. The epidemiological and clinical–pathological features of these patients are reported in Table 1 (central column).

### Group 2: patients with normal adrenal function detected at the first ACTH test

Thirty-five patients (63.6%) showed normal levels of basal cortisol and ACTH and a normal response to the first short ACTH test. These results demonstrated that, at the time of the stimulation test, the adrenal glands were normally functioning despite the ongoing TKI treatment. The epidemiological and clinical–pathological features of these patients are reported in Table 1 (right column).

**Fig. 1:**
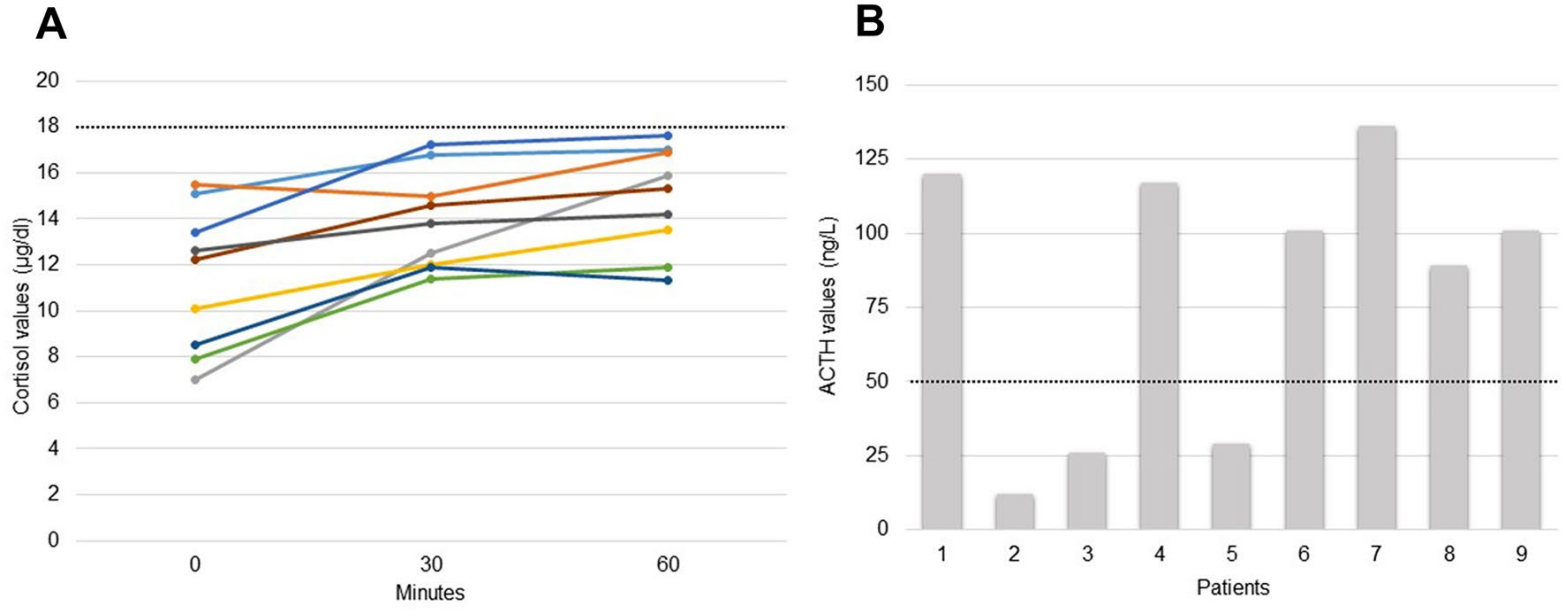
Panel **A** Serum basal and ACTH-stimulated cortisol in the nine patients who developed primary adrenal insufficiency during follow-up and with a previous normal ACTH stimulation test. The diagnosis of adrenal insufficiency was defined by a peak cortisol level below 18 µg/dL at either 30 or 60 min. Panel **B** ACTH values at the time of the normal ACTH stimulation test in the same nine patients of panel A: 6/9 had already elevated values of basal ACTH (> 50 ng/L, as indicated by the dashed line), thus precluding imminent AI subsequently demonstrated by a blunted ACTH test

9/35 (25.7%) patients of Group 2 developed AI during TKI treatment as assessed by a blunted response of the cortisol to a subsequent short ACTH stimulation test (cortisol peak—median: 15.3 µg/dL; mean: 14.9 µg/dL; range: 11.9–17.6 µg/dL) (Fig. 1, panel A). The median and mean time elapsing from the initiation of the therapy and the evidence of hypocortisolism was 11 months and 28.8 months, respectively. In six out of nine (66.7%) patients, the basal ACTH levels ​​were above the maximum normal value already at the first ACTH test despite the normal response of the cortisol to the stimulus (basal ACTH median: 109 ng/L; mean: 110.7 ng/L; range: 89–136 ng/L; nv: < 50 ng/L) (Fig. 1, panel B). Epidemiological, clinical, and pathological features of these patients are reported in Table 2.

**Table 2:** Epidemiological, clinical and pathological features of patients treated with TKI and who developed adrenal insufficiency during follow-up

Total pts (*n* = 9)		
Characteristics	No.	%
Sex		
Male Female	6 3	66.7 33.3
Age at diagnosis, years		
Mean Median Range	53.4 52.0 39–67	
Age at screening, years		
Mean Median Range	60.3 63.0 39–73	
Histology		
PTC FTC MTC PDTC	2 3 1 3	22.3 33.3 11.1 33.3
TKI at ACTH test		
Lenvatinib Vandetanib Cabozantinib	7 1 1	77.8 11.1 11.1
Lines of TKI		
1 2 4	5 3 1	55.6 33.3 11.1

The other 26 patients (74.3%) are still on follow-up without evidence of AI after a median and mean time from the initiation of the TKI therapy of 33 months and 58.7 months, respectively.

### Elapsing time from initiation of TKI and evidence of adrenal insufficiency

The 35 patients who had a first normal short ACTH test were submitted to subsequent stimulation tests every 6 months. Considering the time between the starting date of TKI and the first abnormal ACTH test in the 9 patients who developed AI, we found that in 5/9 (55.6%) patients the AI appeared within 12 months, in 2/9 (22.2%) patients between 12 and 36 months and in 2/9 (22.2%) patients after 36 months (range 6–103 months).

### Evaluation of adrenal insufficiency in our patients

A total of 29 out of 55 (52.7%) patients of our series developed a subclinical hypocortisolism while treatment with TKI. At the time of the diagnosis of AI, all patients, including those who were already affected (*n* = 20) and those who developed hypocortisolism during follow-up (*n* = 9), showed normal values of serum sodium (median: 140 mEq/L; mean: 140.2 mEq/L; range: 135–144 mEq/L). Only few of them (*n* = 3) had low levels of serum potassium and none had elevated values (median: 3.8 mEq/L; mean: 3.7 mEq/L; range: 2.8–4.5 mEq/L). All patients with AI showed normal values of blood pressure, although many of them were under antihypertensive drugs. Nobody showed hypotension.

The adrenal antibodies were evaluated in all hypocortisolemic patients and were negative in all cases. The evaluation of the morphology of the adrenal glands by reviewing the CT scans performed during the follow-up did not show any significant alteration such as cortical congestion, hemorrhage, or necrosis in any patient. As previously said, other causes of corticotropic axis insufficiency were evaluated and excluded before enrolling the patients in the present study.

The clinical evaluation of our patients indicated that 23/29 (79.3%) patients with AI complained of fatigue (12 patients—Grade 1, 8 patients—Grade 2, 3 patient—Grade 3, according to CTCAE) before starting glucocorticoid therapy. All patients were treated with cortisone acetate at a daily dose of 25–37.5 mg daily and this symptom was re-evaluated during follow-up, resulting in improvement in 78.3% of the patients (7 patients—Grade 0, 13 patients—Grade 1, 3 patient—Grade 2, according to CTCAE). The ACTH values monitored during glucocorticoid therapy showed that this parameter remained elevated during replacement therapy.

### Prognostic factors of primary adrenal insufficiency in thyroid cancer patients treated with TKI

As shown in Table 3, the comparison of different clinical, epidemiological and pathological features between patients with (*n* = 29) (Group A) and without hypocortisolism (*n* = 26) (Group B) did not show any significant correlation between the development of AI and the main clinical, epidemiological and pathological features.

**Table 3:** Epidemiological and clinical–pathological features of patients (Group A vs Group B)

Characteristics	Total pts (*n* = 55)		Group A (*n* = 29)		Group B (*n* = 26)	
	No.	%	No.	%	No.	%
Sex						
Male Female	39 16	70.9 29.1	22 7	75.9 24.1	17 9	65.4 34.6
Age at diagnosis, years						
Mean Median Range	49.2 47 13–68		49.6 47.0 34–67		48.0 46.5 13–68	
Age at screening, years						
Mean Median Range	55.2 58 20–73		56.0 56.5 34–73		53.4 56.5 20–73	
Histology						
PTC FTC MTC PDTC	12 11 24 8	21.8 20.0 43.6 14.6	8 7 9 5	27.6 24.1 31.0 17.3	4 4 15 3	15.4 15.4 57.7 11.5
TKI at ACTH test						
Lenvatinib Vandetanib Cabozantinib	30 22 3	54.5 40.0 5.5	19 9 1	65.5 31.0 3.5	11 13 2	42.3 50.0 7.7
Lines of TKI						
1 2 4	45 9 1	81.8 16.4 1.8	24 4 1	82.7 13.8 3.5	21 5 0	80.8 19.2 0

**Fig. 2:**
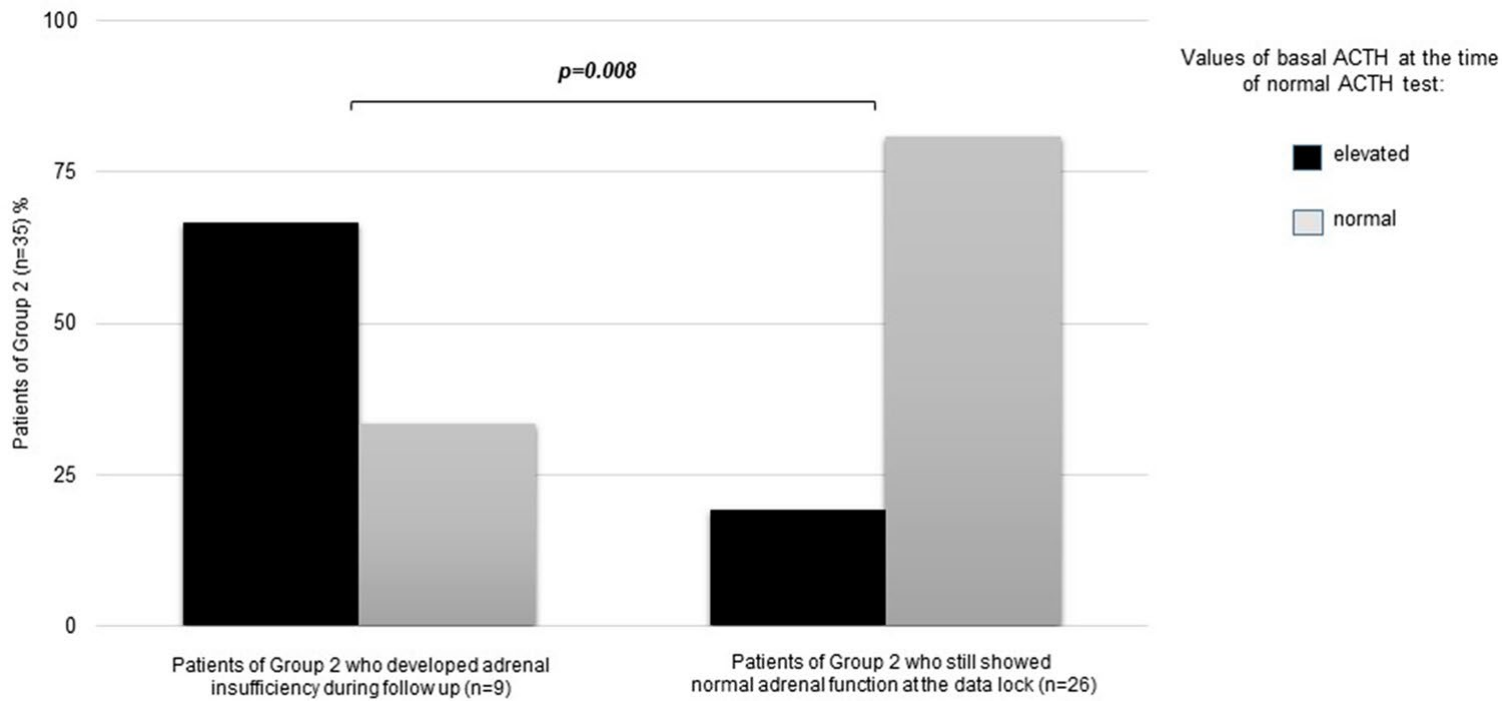
Prevalence of cases with elevated basal level of ACTH (mean 95 ng/L; median 101 ng/L, range 54–136 ng/L) at the time of the first normal ACTH stimulation test during TKI treatment, both in patients who further developed adrenal insufficiency (*n* = 9) and those who did not (*n* = 26). A significant higher number of cases with elevated ACTH was observed in the first group

Looking at the serum basal ACTH levels at the time of the first normal stimulation test (Group 2), we observed that abnormal elevated ACTH values (mean 95 ng/L; median 101 ng/L, range 54–136 ng/L) were significantly more frequent in patients who developed AI during follow-up (i.e., 6/9 patients) respect to those who had a still normal adrenal function at the time of data lock (Fig. 2).

## Discussion

Tyrosine kinase inhibitors represent the most efficacious treatment in locally advanced or metastatic thyroid cancer [7, 20]. Lenvatinib and sorafenib are multitarget inhibitors approved for the treatment of progressive locally advanced or metastatic radioiodine-refractory thyroid cancer [2, 3]. Vandetanib and cabozantinib are the multitarget drugs approved for the treatment of locally advanced or metastatic MTC [4, 5, 21, 22].

Patients treated with TKI can develop important AEs such as hypertension, fatigue, nausea, anorexia, weight loss and diarrhea during follow-up, which must be promptly treated and, in severe cases, can require a reduction/interruption of TKI [9, 23, 24]. Among the AEs evaluated during TKI treatment, the monitoring of adrenal function is not routinely performed, but recently an impact of vandetanib and lenvatinib on the cortisol production has been reported in a relatively small series of patients treated for advanced thyroid cancers [16, 17]. One could argue that these types of patients who have an advanced and progressive cancer could develop hypocortisolism for several reasons including depression, comorbidities, and psychological impairment for the consciousness of the severity of disease, but we excluded all these causes before starting the study. Although the pathogenetic mechanism of AI is still poorly understood, the most reliable hypothesis is that TKI may induce an adrenal damage through a severe alteration of physiologic angiogenesis [25].

In this study, we evaluated the adrenal function in 55 thyroid cancer patients during TKI therapy. We analyzed the adrenal function of our patients by measuring basal cortisol and ACTH and using the short ACTH stimulation test and we found that 29/55 (52.7%) patients treated with different types of TKI had a subclinical AI. This prevalence is in line with that found by Colombo et al. who found that 6/12 (50%) patients treated with either vandetanib or lenvatinib showed a blunted cortisol response to ACTH stimulation test [16]. Similarly, Monti et al. found that 7/13 (53.8%) patients under treatment with lenvatinib had hypocortisolism when investigated with an ACTH stimulation test [17]. Our study confirmed, in a much bigger series of patients and including cases treated not only with vandetanib or lenvatinib, but also with cabozantinib that more than half of these patients can develop subclinical AI. The discovery of this subclinical disease in a patient treated with TKI is of clinical relevance, because it requires immediate treatment [18].

One relevant finding of our study is that AI needs to be searched for when still subclinical, since there are no symptoms or signs that can alert about its presence, as demonstrated by the normal values of electrolytes and blood pressure in all our patients (*n* = 29) with AI, even in those who were already affected at the time of the first ACTH test. Even fatigue, which is a common AE of the TKI therapy, cannot be considered as a symptom of the AI as demonstrated by the evidence that not all patients with AI reported this symptom and, when reported, it was sometimes of very low grade. For these reasons, the diagnosis of AI and the following therapy cannot be done only on clinical judgment, but by a regular monitoring of basal cortisol and ACTH and performing ACTH stimulation test. Regarding the basal value of ACTH, our results clearly demonstrated that, when elevated, it is a prognostic factor of AI, but also some patients with normal basal level of ACTH had a positive stimulation test indicating the presence of AI. Since the positive test is already diagnostic of AI [18], our findings suggest performing the test in all patients taking TKI, even if basal cortisol and ACTH are in the normal range and during the follow-up for early detection of AI.

Nine patients developed AI during the follow-up at different times from the initiation of TKI therapy. It is worth noting that some of these patients developed AI after 3 years of TKI treatment, thus indicating that the possibility of developing this AE must be taken into consideration even after years from the beginning of the TKI therapy and that the test must be performed periodically.

At variance with Monti et al, in our series the advanced age was not predisposing to AI as well as it wasn’t for the histotype and the type of TKI. In agreement with the finding of Monti et al. we found that a prognostic factor for the possibility to develop AI is the abnormal elevated levels of ACTH, even if in the presence of normal basal levels of cortisol. Indeed, the biochemical and clinical course of primary AI is characterized by a progressive reduction of cortisol production, followed by a compensatory increase of ACTH and then an overt hypocortisolism [18]. In our series, a subset of 5/26 (19.2%) patients, who have not yet developed AI despite the relatively long-term follow-up (median: 33 months, mean: 58.7 months), showed elevated values of basal ACTH and we cannot exclude that they would develop AI in the near future. These patients must be monitored periodically for the risk to develop AI. Nevertheless, a certain percentage of cases, 33.3% in our series, were diagnosed only with the stimulation test that remains the gold standard test to define the diagnosis of AI as reported by the AI guidelines [18].

Regarding the primary AI, one could argue that patients could be affected by other adrenal diseases. We excluded the presence of adrenal antibodies that could be responsible for Addison’s disease [18]. The same results were obtained in the previously reported series [16, 17]. Moreover, we also performed the morphological evaluation of the adrenal glands that did not show any sign of cortical congestion, hemorrhage or necrosis that, according to the results of studies performed in rats and monkeys treated with sunitinib [15], could be expected in patients treated with TKI. According to these data, we can strongly support the hypothesis of a correlation of the AI with the TKI treatment, even if we do not know the exact mechanisms underlying the disease. Nevertheless, we can suppose that, as it happens for other AEs, the strong anti-VEGF-R activity of these drugs could be responsible for AI [10]. If this is the case, it is likely that AI will not develop in patients treated with anti-RET or anti-TRK-specific inhibitors such as selpercatinib, pralsetinib and larotrectinib [26–28].

All our patients with primary AI were treated with cortisone acetate and we observed an improvement of fatigue in most of them, but as previously said this symptom can be due also to other causes and cannot be used to establish if the glucocorticoid therapy is adequate. Moreover, the evaluation of ACTH values during glucocorticoid therapy showed that these values remained elevated, confirming that the ACTH values cannot represent a marker to be used for adjusting the replacement therapy [18]. Nevertheless, replacement therapy with corticosteroid should be done to avoid the potential adrenal crisis that can happen in these patients when facing an unexpected physical or psychological stress [18].

In conclusion, AI is a frequent AE of TKI therapy, at least in advanced thyroid cancer patients, which can arise during treatment even after years of therapy. It is frequently asymptomatic and, for this reason, it should be investigated by monitoring not only the basal levels of both cortisol and ACTH and verified, but with an ACTH stimulation test, especially in the absence of elevated values of basal ACTH. The early detection of AI allows to immediately start glucocorticoid replacement therapy, whose main aim is to reduce the risk of an adrenal crisis and at the same time improve, at least in part, the level of fatigue mainly determined by TKI treatment.

## Data Availability

Some or all datasets generated during and/or analyzed during the current study are not publicly available, but are available from the corresponding author on reasonable request.
